# Measures of kidney function by minimally invasive techniques correlate with histological glomerular damage in SCID mice with adriamycin-induced nephropathy

**DOI:** 10.1038/srep13601

**Published:** 2015-09-02

**Authors:** Lauren Scarfe, Aleksandra Rak-Raszewska, Stefania Geraci, Darsy Darssan, Jack Sharkey, Jiaguo Huang, Neal C. Burton, David Mason, Parisa Ranjzad, Simon Kenny, Norbert Gretz, Raphaël Lévy, B. Kevin Park, Marta García-Fiñana, Adrian S. Woolf, Patricia Murray, Bettina Wilm

**Affiliations:** 1 Department of Cellular and Molecular Physiology, Institute of Translational Medicine, University of Liverpool, Liverpool, UK; 2 Department of Biostatistics, Institute of Translational Medicine, University of Liverpool, Liverpool, UK; 3 Department of Molecular and Clinical Pharmacology, Institute of Translational Medicine, University of Liverpool, Liverpool, UK; 4 Centre for Cell Imaging, Institute of Integrative Biology, University of Liverpool, Liverpool, UK; 5 Medical Research Centre, Medical Faculty Mannheim, University of Heidelberg, Mannheim, Germany; 6 iThera Medical, Munich, Germany; 7 Centre for Paediatrics and Child Health, Institute of Human Development, Faculty of Medical and Human Sciences, University of Manchester, Manchester, UK

## Abstract

Maximising the use of preclinical murine models of progressive kidney disease as test beds for therapies ideally requires kidney function to be measured repeatedly in a safe, minimally invasive manner. To date, most studies of murine nephropathy depend on unreliable markers of renal physiological function, exemplified by measuring blood levels of creatinine and urea, and on various end points necessitating sacrifice of experimental animals to assess histological damage, thus counteracting the principles of Replacement, Refinement and Reduction. Here, we applied two novel minimally invasive techniques to measure kidney function in SCID mice with adriamycin-induced nephropathy. We employed i) a transcutaneous device that measures the half-life of intravenously administered FITC-sinistrin, a molecule cleared by glomerular filtration; and ii) multispectral optoacoustic tomography, a photoacoustic imaging device that directly visualises the clearance of the near infrared dye, IRDye 800CW carboxylate. Measurements with either technique showed a significant impairment of renal function in experimental animals *versus* controls, with significant correlations with the proportion of scarred glomeruli five weeks after induction of injury. These technologies provide clinically relevant functional data and should be widely adopted for testing the efficacies of novel therapies. Moreover, their use will also lead to a reduction in experimental animal numbers.

Chronic kidney disease (CKD) represents a huge socioeconomic burden, with treatment options for severe disease being limited to dialysis and transplantation. By developing regenerative medicine therapies (RMTs) for CKD in preclinical models, novel and alternative clinical approaches may be established to halt progression to end stage renal disease (ESRD). In order to maximise the use of preclinical murine models of progressive kidney disease as experimentation platforms for novel therapies, including drugs and stem cells^1^,^2^,^3^,^4^, kidney function, in concert with structural morphology, needs to be monitored repeatedly in a safe, minimally invasive manner. The glomerular filtration rate (GFR) is considered the gold standard measure of the main physiological function of the kidney, i.e. to efficiently clear small molecular waste products from the blood stream. However, its measurement generally requires repeated blood sampling, and for some methods, precisely timed urine collections. While these techniques are feasible in patients, it is much more cumbersome and challenging to apply them to small experimental animals. To date, therefore, most mouse studies have used surrogate markers of GFR, exemplified by measuring blood levels of creatinine and urea. These are unreliable measures of GFR because levels only rise when over 50% of renal function is lost; moreover, factors such as food intake can affect blood levels independently of GFR. Furthermore, most studies of murine nephropathy have relied heavily on end point analysis for the assessment of histological damage, which necessitates killing experimental animals. While this provides useful data, the sole reliance on necropsy material is not compatible with optimal application of the principles of Replacement, Refinement and Reduction (the 3Rs).

Here, we have evaluated the ability of two minimally invasive *in vivo* technologies to monitor kidney function in an adriamycin (doxorubicin)-induced nephropathy mouse model. The anthracycline antibiotic adriamycin (ADR) primarily targets both glomerular endothelium and podocytes in rodents and is a model of focal segmental glomerulosclerosis (FSGS), which accounts for 2.3% of all cases of ESRD^5^. The incidence of primary FSGS has increased by up to 13 fold in the last 30 years^6^. Along with diabetic nephropathy, it is a common cause of severe proteinuria. Administration of a single dose of ADR in both BALB/c and BALB/c severe combined immunodeficient (SCID) mice leads to two phases of disease^4^,^7^,^8^,^9^. In the first, glomeruli are grossly histologically intact (i.e. they show no scarring) but their ability to act as a macromolecular barrier is severely compromised. This is manifest as ‘proteinuria’. Its magnitude can be assessed by measuring the amount of albumin, which appears in the urine over unit time; the simpler measure, the urine albumin/creatinine ratio, can also be employed. In the second phase of disease, glomeruli become scarred or ‘sclerotic’ and this leads to the loss of total filtration surface. This occurs long after ADR has been cleared and it exemplifies the ‘progression of CKD’ often observed in clinical practice. This has two clinical outcomes: both the degree of proteinuria may fall and, at the same time, the GFR also falls. Given that an unchecked fall of GFR will ultimately lead to ESRD, minimally invasive measures which correlate with gross structural glomerular damage would be highly informative.

Importantly, we chose SCID mice in the current study with a longer-term view of using immunocompromised mice with drug-induced nephropathy as test beds for assessing the safety and efficacy of different types of human stem cells. The two minimally invasive technologies comprised firstly, a transcutaneous electronic device for measuring the clearance from the blood of (FITC)-sinistrin, a molecule of approximately 4000 Da cleared by glomerular filtration^10^,^11^. This device can be applied in conscious animals, which is a key advantage, given the well-described confounding effects of general anaesthesia on GFR^12^,^13^. The second technology was multispectral optoacoustic tomography (MSOT). This imaging technology was used to measure the passage of a near infra-red dye, IRDye 800CW carboxylate (from now on referred to simply as IRDye), through the kidney parenchyma into the renal pelvis. Specifically, a recent report suggested that MSOT could effectively monitor renal clearance of IRDye in mice^14^. MSOT illuminates tissue with light pulses at multiple wavelengths and detects acoustic waves generated by the thermoelastic expansion which follows light absorption^15^,^16^. The technique is not harmful and offers very good temporal and spatial resolution in live animals; however, it requires the animals to be studied under general anaesthesia.

Results obtained with these techniques were compared with standard biochemical and histological indicators of kidney damage. This included 24-hour-albuminuria or urinary albumin-to-creatinine ratio, serum creatinine (SCr), blood urea nitrogen (BUN), and Picro-Sirius red (PSR) and Masson’s Trichrome staining in order to detect structural histological damage. Subsequently, we applied an extensive range of statistical tests to determine whether the transcutaneous device and MSOT are appropriate tools for monitoring kidney function. Furthermore, we assessed whether both technologies could be used to predict histological damage during progression to CKD in the ADR model.

## Results

### The half-life of transcutaneously measured FITC-sinistrin becomes significantly increased over the course of ADR-induced nephropathy

**Figure 1:**
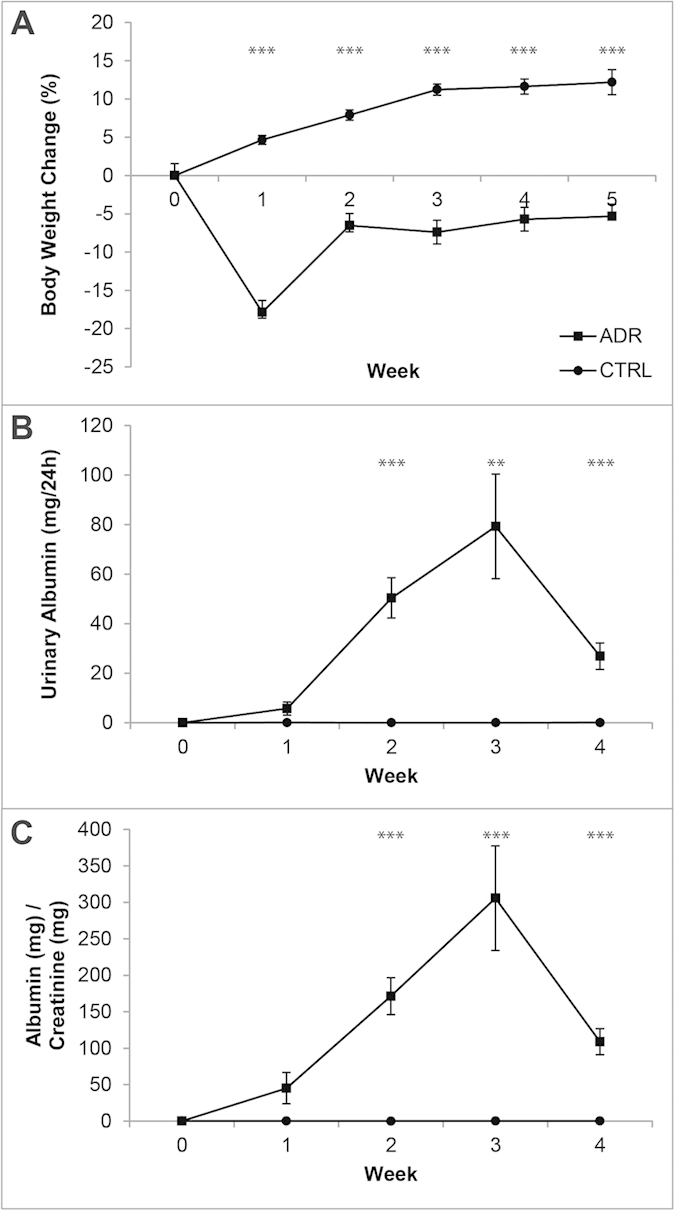
(**A**) Mean change in body weight of mice up to five weeks after adriamycin administration. Animals of the ADR group had a mean body weight loss of approximately 20% by week 1, while control animals had a mean body weight gain of 5%. (**B**) Mean albuminuria over 24 hours, measured weekly. ADR-administered animals had a mean maximum of albuminuria of approximately 90 mg/24 h at 3 weeks, while in control animals urinary albumin stayed constant throughout the study at around 0.03 mg/24 h. (**C**) Mean albumin:creatinine ratio (alb:cr) measured weekly, showing similar temporal dynamics to 24 h albuminuria. Data points (circles = control, *n* = 5; squares = ADR-administered, *n* = 6) and bars show mean ± standard error. Asterisks indicate significance of mixed-design ANOVA models: p ≤ 0.01 (**), p ≤ 0.001 (***), see also Table S3.

Kidney injury was induced by intravenous (iv) ADR administration once in 6 SCID female mice while 5 control SCID females received saline injection. The weights of ADR-administered *versus* control mice reached their lowest point one week after nephrotoxin administration. Over the next four weeks, weights in the ADR group returned towards time-matched control values but did not attain them (Fig. 1A, S1A). To determine damage to the glomerular macromolecular barrier, we measured total 24-hour urinary excretion and the albumin:creatinine ratio on a weekly basis (Fig. 1B,C, S1B,C). Using either measure, albuminuria was significantly elevated in the ADR group at weeks 2 to 4, with attenuation at week 4 (Fig. 1B,C, Table S1A, S3). Similar effects have been previously reported in ADR-induced SCID mice^4^,^9^,^17^. A linear mixed-effects (LME) model showed statistically significant linear and quadratic changes of albuminuria over time within the ADR group (p < 0.001 for both the linear and second degree terms, Table 1).

**Figure 2:**
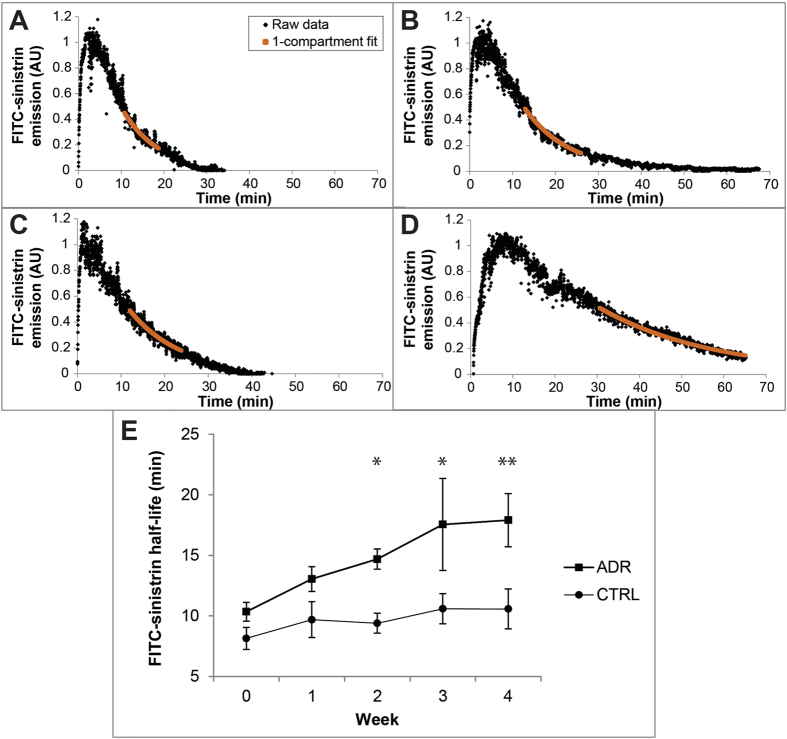
(**A–D**) Typical FITC-sinistrin kinetic curves (arbitrary units) in a control mouse at week zero (**A**), and four (**B**); and in an ADR-administered mouse prior to (**C**) and at four weeks post-adriamycin administration (**D**). (**E**) The mean half-life of FITC-sinistrin for the ADR-administered and control groups is shown weekly for four weeks. Data points (circles = control, *n* = 5; squares = ADR-administered, *n* = 6) and bars show mean ± standard error. Asterisks indicate significance of mixed design ANOVA models: p ≤ 0.05 (*), p ≤ 0.01 (**), see also Table S3.

To assess changes in glomerular filtration over time with the transcutaneous device, we undertook serial measurements of FITC-sinistrin half-life over 4 weeks. Representative examples of clearance curves from control and ADR animals are shown in Fig. 2A–D. The FITC-sinistrin half-life in the ADR group was increased with statistical significance from week 2 onwards (Fig. 2E, Table S1A, S3). Given that GFR is inversely correlated to the FITC-sinistrin half-life^10^,^18^, these results suggest that GFR is slightly impaired 2 weeks after ADR administration and deteriorates further between weeks 3 and 4, reflecting the progressive nature of the nephropathy^19^. An LME model revealed statistically significant linear changes in the FITC-sinistrin half-life within the ADR group over time, with an average increment of 2.45 (=(0.02 + 0.33) × 7) minutes per week (p < 0.001). These changes were statistically different when compared with controls, in which no significant changes in half-life were detected (p = 0.76, Table 1). The levels of BUN and SCr as indicators of kidney function revealed no significant differences between the experimental control groups at week 5 (Figure S1D,E).

### The passage of IRDye through the kidney measured using MSOT is delayed in ADR mice

**Figure 3:**
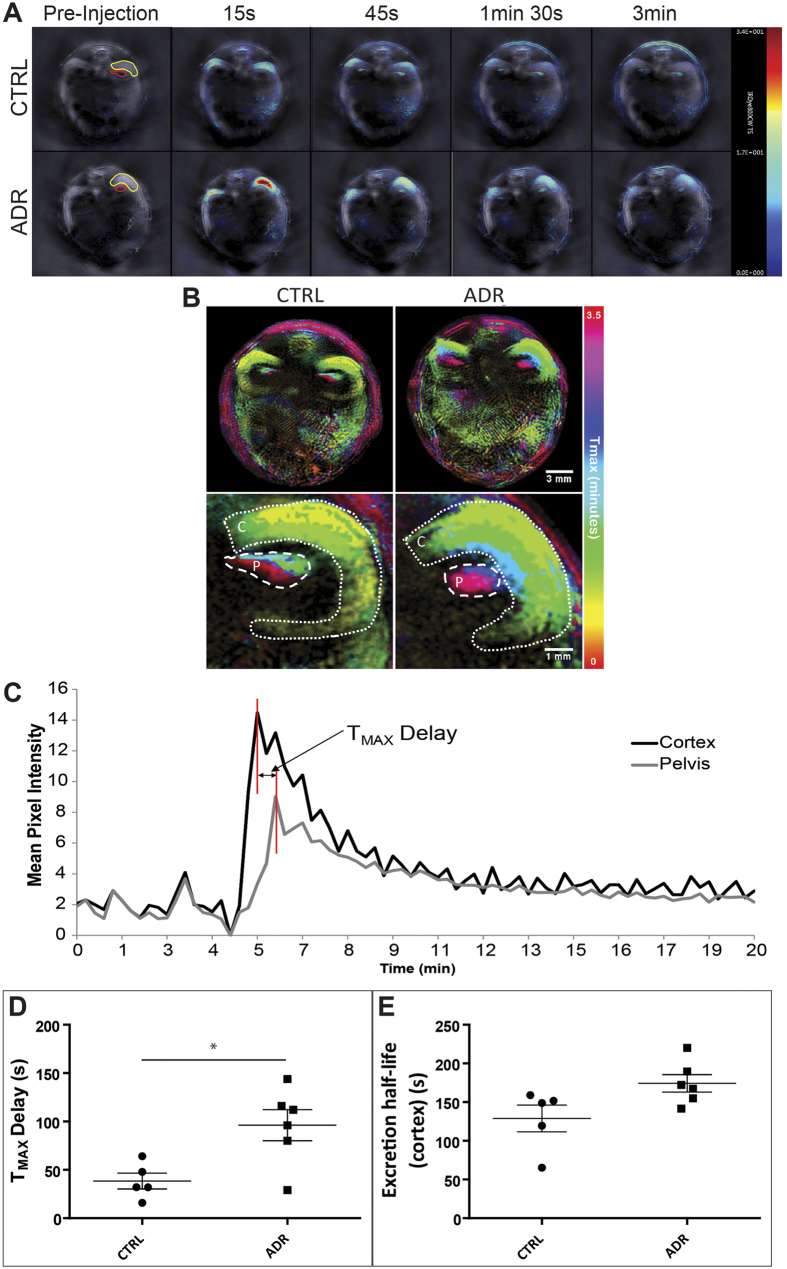
(**A**) MSOT images of a typical control (top) and ADR-administered (bottom) mouse before and after the administration of IRDye. Region of interests (ROIs) depicted in yellow (cortex) and red (papilla/pelvis region). Times indicated above apply to both control and ADR-treated animal. (**B**) Temporal colour maps of control and ADR-administered mice reveal a delay in IRDye clearance kinetics in the treated mice. (**C**) Characteristic plot of mean pixel intensity (MSOT arbitrary units) from ROIs drawn around the renal cortex (black) and papilla/pelvis region (grey). (**D**,**E**) Graphs showing distribution of T_MAX_ delay (**D**) and the excretion half-life in the cortex (**E**) in ADR-administered and control animals. Data points (**D**,**E**) represent individual animals (circles = control, *n* = 5; squares = ADR-administered, *n* = 6) and lines represent mean ± standard error. Asterisks indicate significance of two-sample t-tests: p ≤ 0.05 (*), see also Table S2.

To investigate the potential of MSOT for evaluating renal function in ADR mice, IRDye clearance was monitored in real-time at week 5, before killing for necropsy studies. Movies and snap-shot images taken prior to, and 15 s, 45 s, 1 min 30 s and 3 min after administration of IRDye showed that its passage through the kidney appeared to be delayed in ADR *versus* control mice (Fig. 3A, supplementary movie 1, 2). More specifically, temporal colour maps of the clearance kinetics confirmed that in nephropathic mice, the dye took longer to transit from the kidney cortex to the papilla/pelvis region than in controls (Fig. 3B). Visualisation of the T_MAX_ allows discrimination between the ADR and the control group, highlighted by the peak signal intensity in the cortex of the control animals appearing at ~30 s and visualised in yellow. By contrast, the yellow tone indicating 30 s peak signal intensity is absent in the ADR treated mice; instead, green colour tones visualise peak signal intensities that appear with a delay at around 1 min. Furthermore the papilla/pelvis region of control animals show peak concentrations from 1.5–3 minutes (green, blue, violet, red), whereas the ADR treated mice show peak concentrations in this region only after 3 minutes (violet, red, Fig. 3B). We determined the pharmacokinetics of IRDye clearance by calculating the T_MAX_ delay between the renal cortex and pelvis (Fig. 3C,D, Table S1B), and the clearance half-life in the cortex, using exponential decay fitting (Fig. 3C,E, Table S1B). T_MAX_ delay was significantly increased in the ADR-administered group *versus* controls (p = 0.015), and there was a non-significant increase in clearance half-life between these two groups (Fig. 3D,E; Table S2). However, equilibrium dialysis revealed that over 40% of IRDye bound to plasma proteins (Table S4), suggesting that the exponential decay might be shorter than expected in ADR mice due to leakage of plasma proteins through the glomerular filtration barrier, thus leading to a potential underestimation of the true clearance half-life in the cortex.

### Minimally invasively measured clearance kinetics of sinistrin and IRDye show a strong correlation with glomerular histological damage

**Figure 4:**
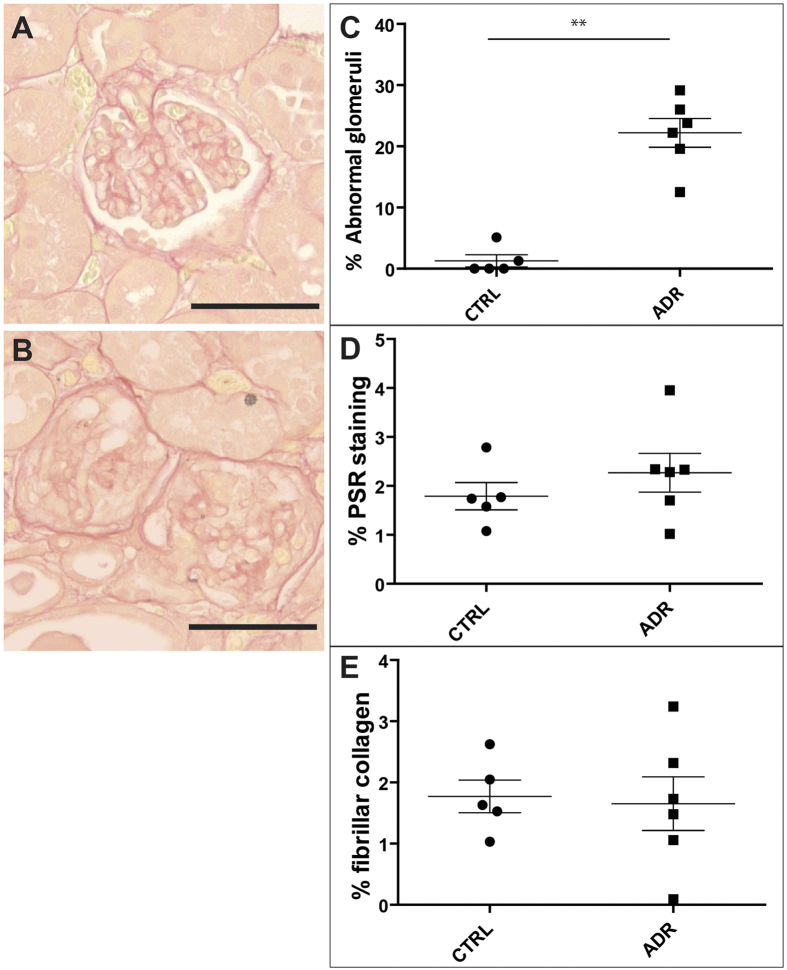
Typical examples of normal (A) and abnormal (B) glomeruli imaged under bright field microscopy in 4 μm Picro-Sirius Red-stained paraffin sections at week five. On one kidney section per animal the percentage of abnormal glomeruli was determined (**C**). One whole kidney section per animal was photographed under bright field and polarised microscopy and the images were stitched together to produce one whole kidney image. On each whole-kidney image the percentage of PSR staining under bright field light (**D**) and the percentage of fibrillar collagen under polarised light (**E**) were quantified using image analysis software. Scale bars (**A**,**B**) represent 50 μm. Data points (**C**,**E**) represent individual animals (circles = control, *n* = 5; squares = ADR-administered, *n* = 6) and lines represent mean ± standard error. Asterisks indicate significance of two-sample t-tests: p ≤ 0.01 (**), see also Table S2.

To visualise and measure structural damage, we made use of Picro-Sirius Red (PSR) staining which binds specifically to fibrillar collagen type I and III under polarising microscopy. Analysing PSR staining on parasagittal kidney sections from mice necropsied at week 5, we determined that ADR kidneys contained a subset (12–29%, across the whole experimental group) of glomeruli with lesions (Fig. 4B,C; Tables S1B, S2). In these glomeruli, there was a loss of capillary loops and extension of PSR staining into the glomerular tuft beyond the normal tree-like mesangial pattern. In control kidneys, such glomerular lesions were rarely detected (Fig. 4A). Quantification of total PSR staining (i.e. not confined to glomeruli) under bright field microscopy, and of fibrillar collagen under polarising microscopy, failed to show significant differences in ADR *versus* control kidneys (Fig. 4D,E, S2A,B; Tables S1B, S2). However, we noted the occurrence of proteinaceous casts together with flattened tubular epithelia in both PSR- and Masson’s Trichrome-stained ADR kidneys viewed under bright field microscopy (Figure S2B,D), but not in control kidneys (Figure S2A, C). These observations show that in this model, at this particular time point, glomerular damage is the predominant lesion, while tubulo-interstitial changes are much less prominent.

**Figure 5:**
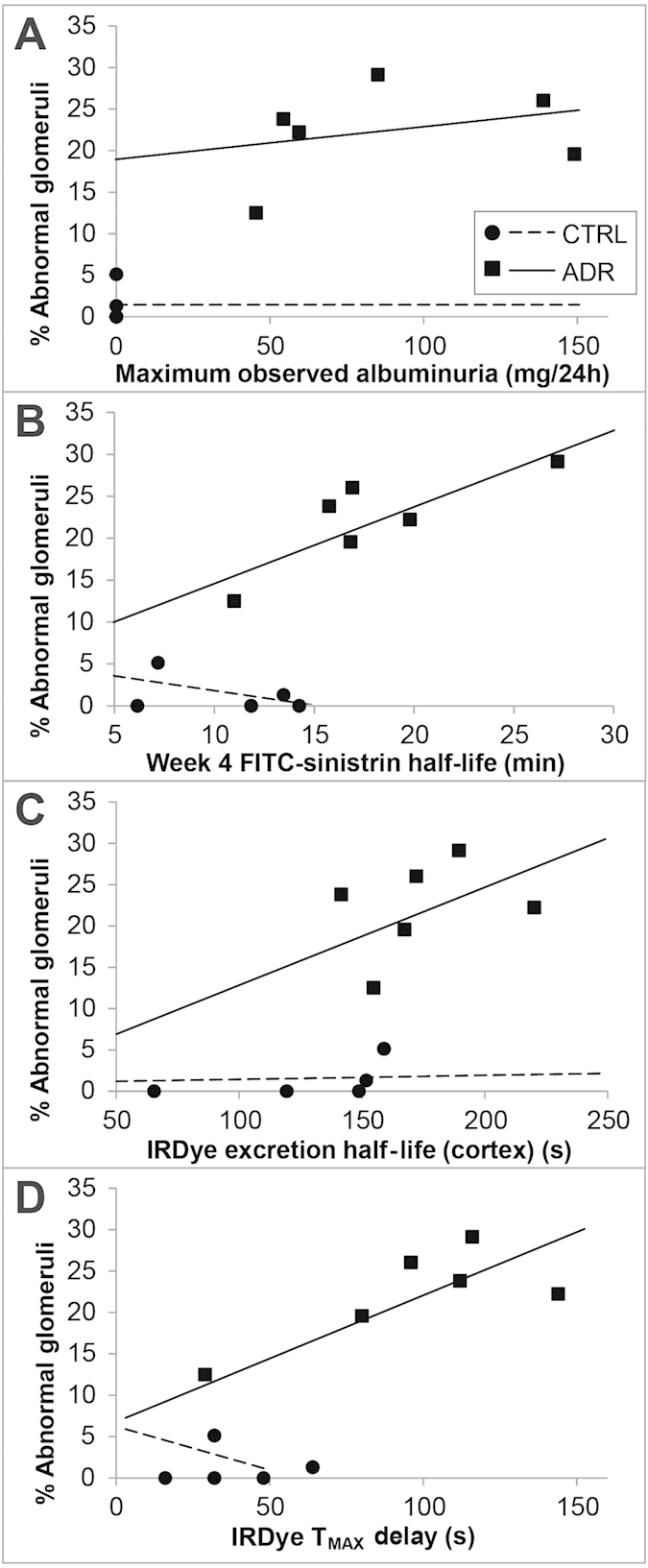
Correlation graphs showing the correlation of both control and ADR group data between the percentage of abnormal glomeruli and maximum observed albuminuria (A), FITC-Sinistrin half-life at week 4 (B), IRDye excretion half-life in the cortex at week 5 (C) and IRDye T_MAX_ delay (D) at week 5. Data points represent individual animals (circles = control, *n* = 5; squares = ADR-administered, *n* = 6). Trendlines for control (dashed line) and ADR-administered (solid line) animals are displayed.

Next, we evaluated whether the two novel minimally invasive dye clearance methods we have used here to detect kidney filtration function, are suitable for long-term regenerative medicine therapy studies through their statistical association with detected glomerular damage. Importantly, since we had observed a wide range in the proportions of scarred glomeruli (as assessed by the above criteria) in the ADR kidneys, we questioned whether specific glomerular damage in individual mice was associated with specific measures of kidney function. Therefore, we examined the relationship in individual animals between glomerular histological lesions and (i) albuminuria, (ii) FITC-sinistrin half-life and (iii) IRDye excretion kinetics in individual animals (Fig. 5). In ADR mice, there was no significant association between glomerular histological damage (week 5) and the amount of albuminuria measured at the maximum (weeks 2–3) or at the final point of urine collection in week 4 (Table 2). Therefore, albuminuria is not a reliable measure of glomerular histological damage in this model. By contrast, assessment of the relationship between FITC-sinistrin half-life and the proportion of histologically damaged glomeruli revealed a significant positive correlation between FITC-sinistrin half-life values at week 4 and glomerular damage at week 5 (coefficient = 0.94, p = 0.001; see Table 2).

When we assessed the relationship at 5 weeks between glomerular histological damage and IRDye clearance half-life, we found a significant association in the ADR group (coefficient = 0.12, p = 0.02; Table 2). Furthermore, the T_MAX_ delay was significantly associated with glomerular histological damage in the ADR group (coefficient = 0.16, p = 0.002; Table 2). The coefficient suggests that an increment of 60 seconds in T_MAX_ delay is associated with an increment of glomerular histological damage of about 10%.

## Discussion

Here, we report for the first time the use of transcutaneous measurement of FITC-sinistrin decay and MSOT detection of IRDye clearance as measures of glomerular filtration function in SCID mice with ADR-induced nephropathy. Our albuminuria measurements strongly suggest that ADR-induced nephropathy featured a loss of glomerular macromolecular barrier integrity in SCID mice from week 2, peaking at week 3, similarly to recently published results^4^,^9^,^17^. In ADR mice, the FITC-sinistrin half-life was significantly prolonged from week 2. The fact that the half-life continued to rise thereafter in the ADR group is a strong indicator of a progressive loss in the ability of the kidneys to excrete small molecules from the circulation, i.e. a progressive decline in GFR. Previous studies have demonstrated the accuracy and reliability of the transcutaneous measurement of FITC-sinistrin half-life as a measure of GFR in various strains of rats and mice^10^,^11^,^18^,^20^,^21^,^22^,^23^. Of note, although remaining elevated, the degree of albuminuria fell between weeks 3 and 4. This again might be explained by a loss in total filtration surface associated with glomerular damage as observed in week 5, consistent with the observed rise in FITC-sinistrin half-life during this period.

Using MSOT imaging, we showed here that IRDye is cleared in a dynamic fashion as it first appears in the kidney cortex and then transits to the renal pelvis, confirming a previous report^14^. The simplest interpretation of the T_MAX_ delay of IRDye clearance we measured in the ADR *versus* control group, is that the glomerular clearance of this small molecule is impaired. The observation that some IRDye binds to plasma proteins, and that the ADR model features proteinuria, may have led to an underestimation of the true ‘T_MAX_ delay’. In addition, it is possible that the passage of IRDye along the lumen of kidney tubules may be compromised in the ADR model but this will require further study.

Our BUN and SCr measurements revealed that these are not useful indicators of ADR-induced nephropathy at a time point when sinistrin clearance deviated most from normal. Both BUN and SCr measurements have been questioned as biomarkers for the early detection of human and rodent kidney disease at the histological level since they frequently identify abnormal kidney function only in the later stages of the diseases^24^,^25^. Previously, an increase in SCr in ADR-induced nephropathy has only been observed in male rodents, while in female rodents, SCr levels remained unchanged when compared to control animals, even though pathohistology clearly indicated the full spectrum of renal damage^3^,^26^,^27^,^28^,^29^,^30^.

Crucially, our observations that BUN and SCr fail to detect significant differences in kidney function at a time point when histological changes are clearly quantifiable, emphasises the need to develop novel molecular biomarkers that are better able to monitor renal health and assess the efficacy of therapeutic interventions, including regenerative medicine therapies^24^,^31^,^32^.

We conclude that transcutaneous measurements of FITC-sinistrin half-life provide a minimally invasive method to repeatedly assess GFR in conscious mice, thus allowing for longitudinal evaluation of kidney function. Furthermore, our analysis suggests that MSOT imaging has the potential to be employed for the repeated and minimally invasive measurement of kidney function in mice by assessing renal clearance of injected small near infrared dyes. The use of near infrared dyes with negligible plasma protein binding properties will further improve MSOT imaging performance for the measurement of renal clearance kinetics. Optoacoustic imaging can also be employed to track administered stem cells labelled with gold nanorods, expressing tyrosinase or near infrared fluorophores in whole animals^33^,^34^,^35^,^36^,^37^,^38^,^39^,^40^. Therefore, this imaging technology will be of high importance for preclinical studies in regenerative approaches to nephropathies as it will allow the detection of labelled cells in parallel with functional measurements of renal clearance kinetics.

In order to assess whether functional kidney data correlated with glomerular scarring, we performed histological analysis of sections by staining with PSR and Masson’s Trichrome. While SCID animals with ADR-induced nephropathy showed a strong elevation in the number of abnormal glomeruli at 5 weeks, at this time point we only observed occasional pathohistological changes in the tubulo-interstitial zone of ADR mice. Cortical glomerular and tubulo-interstitial damage have been reported previously in mice with ADR-induced nephropathy^19^,^26^,^28^,^41^,^42^.

Importantly, we aimed to statistically analyse whether any associations could be detected between glomerular histological damage and albuminuria, FITC-sinistrin half-life or IRDye clearance, respectively, in mice with ADR-induced nephropathy on the SCID background. Our evaluations provide evidence that FITC-sinistrin half-life and T_MAX_ of IRDye clearance were strongly correlated with glomerular scarring in SCID mice with ADR-induced nephropathy. We therefore conclude that FITC-sinistrin half-life and IRDye clearance (T_MAX_) measurements may be good predictors of glomerular histological damage. In contrast, neither peak albuminuria nor albuminuria at 4 weeks after ADR-induced nephropathy were significantly correlated with glomerular scarring, suggesting that urinary albumin measurements fail to provide an accurate reflection of the histological glomerular damage in this model at this time point.

In a recent report, rats with ADR-induced nephropathy were analysed with magnetic resonance imaging to monitor changes in kidney function and histopathology during disease progression. This study is another example, similar to the one we report here, to demonstrate the feasibility of a longitudinal, minimally-invasive imaging technology as an approach to assessment of changes in the kidney over time^43^.

In conclusion, our results indicate that the minimally invasive technologies for detecting FITC-sinistrin half-life and IRDye clearance kinetics could be widely adopted for *in vivo* monitoring of models of RMT in progressive kidney disease. Each technology potentially allows recurrent testing in individual animals, maximising the information obtained as nephropathy and/or regenerative effects progress. The observations that sinistrin clearance and IRDye kinetics significantly correlate with the proportions of damaged glomeruli in individual animals, indicates that these minimally invasive measurements could markedly reduce animal numbers in preclinical models of nephropathy.

## Materials and Methods

### Animals

Female BALB/c severe combined immunodeficient (SCID) mice (Charles River, Margate, UK) were housed in individually ventilated cages at a 12 hour light/dark cycle, with *ad libitum* access to food and water. At age seven to eight weeks, ADR-induced nephropathy was induced in six mice by injecting once intravenously (iv) adriamycin (ADR, doxorubicin hydrochloride, Tocris, Bristol, UK) at 6.3 mg/kg body weight (BW) in 0.9% saline (Braun, Melsungen, Germany), while five control mice received saline. The optimal dose had been previously determined in a dose-finding study, and is similar to previously reported adriamycin dose given to BALB/c SCID mice^4^,^9^,^17^. Mortality in the ADR-administered group during the five-week study period was zero. Experimental animal protocols were performed in accordance with the approved guidelines under a licence granted under the Animals (Scientific Procedures) Act 1986 and approved by the University of Liverpool Animal Ethics Committee. BALB/c SCID mice were used in order to determine parameters for future preclinical regenerative medicine studies in mice with ADR-induced nephropathy.

### Albuminuria, serum creatinine and BUN

To collect urine, mice were housed individually in metabolic cages (Tecniplast, Buguggiate, Italy) once a week for 24 hours (h). Despite being gradually acclimated to the metabolic cages, 24 h urine collection is stressful for mice, leading to weight loss of up to 1.5 g during the time spent in the cages. Because the mice on average weigh less than 20 g, it was necessary to omit urine collection at week five, since the long term anaesthesia for MSOT imaging is another stressful procedure for the mice. Total urine volume was measured and albumin levels were quantified using a Mouse Albumin ELISA Quantification Kit (Bethyl Laboratories, Montgomery, TX, USA) according to manufacturer’s instructions. Urinary creatinine (UCr) was quantified using a plate based colourimetric assay. Blood was collected via cardiac puncture after sacrifice, and separated into serum. Serum creatinine (SCr) and blood urea nitrogen (BUN) were quantified according to manufacturer’s instructions (Detect X Serum Creatinine Detection Kit, Arbor Assays, Ann Arbor, MI, USA; QuantiChrom Urea Assay Kit, BioAssay Systems, Hayward, CA, USA, respectively).

### Histopathology

Kidneys were dissected at week 5, cut along the sagittal plane, and processed for histology using standard methods. Sections (4 μm) were stained with Picro-Sirius Red (PSR, Sigma-Aldrich, Dorset, UK) or Masson’s Trichrome following standard protocols. All glomeruli (on average 116 glomeruli) in one PSR-stained kidney section per animal were blindly scored for glomerular histological damage and the percentage of abnormal glomeruli calculated. Glomeruli were categorised as abnormal if the PSR staining extended beyond the confines of the mesangium into the glomerular tufts. These scarred glomeruli also featured obliteration of capillary spaces and adhesions of tufts to the Bowman capsule. Using one panorama kidney section per animal, fibrillar collagen was detected using polarised microscopy, while PSR-staining in the cortex was captured by bright field microscopy. Images were stitched together^44^ and image analysis software (Fiji) was used for quantification.

### Transcutaneous FITC-sinistrin decay

FITC-sinistrin half-life was measured weekly in all animals^11^. In short, the transcutaneous device (Mannheim Pharma & Diagnostics GmbH, Mannheim, Germany) was fixed to the depilated skin on the back of mice using a double-sided adhesive patch (Lohmann, Neuwied, Germany). Transcutaneous measurement started with background reading one to three min before 0.3 mg/g BW FITC-Sinistrin (Fresenius Kabi, Linz, Austria; diluted in 0.9% saline; Braun, Melsungen, Germany) was administered iv. Animals were allowed to fully recover and move freely until transcutaneous measurement was stopped after 90 minutes (min). Using a 1-compartment model, the half-life of FITC-Sinistrin was calculated from the transcutaneously measured kinetics (Figure S3)^11^.

### Measuring the clearance of IRDye 800CW carboxylate using MSOT

Five weeks after adriamycin or saline administration, anaesthetised (isoflurane) mice had hair removed from the abdominal region and were imaged in the inVision 256-TF MSOT imaging system (iThera Medical, Munich, Germany) using a multispectral protocol for 30 min (rate of 10 frames per second using wavelengths: 700, 730, 760, 775, 785, 800 and 850 nm, and averaging 20 consecutive frames to minimise influences of motion). Five min into the imaging mice received 200 μl (20 nmol) IRDye 800CW carboxylate (LI-COR, USA) in 0.9% saline through a tail vein cannula over a period of 10 seconds (s). Data was reconstructed and multispectral processing performed to resolve signals for the IRDye, including gradient scaling for each animal at a time prior to the injection of the IRDye. Regions of interest (ROIs) drawn around renal cortex and the renal papilla-pelvis region (in short: pelvis) of the right kidney of each mouse were used to determine the time between the mean peak pixel intensity (T_MAX_) in the cortex and the pelvis (T_MAX_ delay). Exponential decay was fitted for mean cortex pixel intensities to determine the characteristic excretion half-life.

To depict the temporal dynamics of IRDye clearance in colour code, a Matlab routine was developed to process a stack of images and calculate a composite image on a pixel-by-pixel basis of T_MAX_, defined as the time (or frame number in the stack) at which the highest intensity was recorded at that pixel. A pseudocolor was applied to display this time (or frame number) per pixel as different colours on the spectrum colour scale. Image stacks were typically composed of 15 images. With a temporal resolution from *in vivo* imaging of approximately 16 s this equated to approximately 4 min of imaging data after injection that were used for temporal analysis. A C_MAX_ image as a maximum intensity projection was computed of the same image stack. ImageJ was used to amplitude modulate the pseudocoloured T_MAX_ image using the C_MAX_ in order to dim areas with little contrast and therefore to reduce background noise in the T_MAX_ caused by an absence of substantial temporal change in a certain pixel.

### Statistical analyses

Linear mixed-effects (LME) models were fitted to characterise changes of albuminuria and of FITC-sinistrin half-life over time. The advantage of using a linear mixed-effects model is that the correlation between measurements across time points within mice is taken into account in a term called the *random term*. Differences in albuminuria and FITC-sinistrin half-life between the treatment and control group (e.g., differences in linear or quadratic changes over time) were subsequently tested using these models.

Multiple linear regression models were applied to test whether glomerular histological damage was associated with transcutaneous measurements of albuminuria (maximum observed values and week 4 values), FITC-sinistrin half-life (week 4 values), T_MAX_ delay and excretion half-life of IRDye (the latter measured at 5 weeks).

Independent t-tests were applied to compare measures of kidney function at 4 weeks and of post-mortem histology measurements at 5 weeks between the control and the treatment group. The assumptions of normality and homogeneity of variances were checked.

### Plasma Protein Binding of IRDye

A dye-protein stock solution was prepared by incubating of 2 ml IRDye (10 μmol) with 8 ml Sprague Dawley rat plasma Li Heparin (Innovative Research, Novi, MI, USA) in phosphate buffered saline (PBS) at 37 °C, while 2 ml PBS was incubated with 8 ml rat plasma as control. Plasma protein-binding measurements were performed by equilibrium dialysis of PBS against dye-protein stock solution (or control stock solution) using a two-chamber dialysis set-up (Equilibrium Dialyzer, Havard Apparatus, Holliston, MA, USA)^45^,^46^. After 24 h the absorption of IRDye in PBS and plasma were determined in three independent measurements by absorption spectroscopy in a microplate reader (Tecan Infinite M200) and the concentrations of IRDye was calculated on the basis of the corresponding molar absorption coefficients (Figure S4, Table S3).

Plasma protein binding (PPB) of IRDye in percent was determined by averaging three independent measurements and following the equation of Beer-Lambert law:


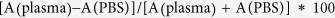


where A is denoted as corresponding to UV absorption.

## Additional Information

**How to cite this article**: Scarfe, L. *et al.* Measures of kidney function by minimally invasive techniques correlate with histological glomerular damage in SCID mice with adriamycin-induced nephropathy. *Sci. Rep.* **5**, 13601; doi: 10.1038/srep13601 (2015).

## Supplementary Material

Supplementary Information

Supplementary Movie 1

Supplementary Movie 2

## Figures and Tables

**Table 1:** Statistical mixed-effects models that describe albuminuria and FITC-Sinistrin half-life over time.

ALBUMINURIA (mg/24 h)				FITC-SINISTRIN HALF-LIFE (min)			
Changes over time (LME)				Changes over time (LME)			
Term	Coef. estimate	SE	P-value	Term	Coef. estimate	SE	P-value
Group	−12.94	8.11	0.12	Intercept (min)	9.96	0.91	<0.001
Group × Time (day)	8.34	1.3	<0.001	Time (day)	0.02	0.07	0.76
Group × Time^2^ (day^2^)	−0.24	0.04	<0.001	Time × Group (day)	0.33	0.07	<0.001
Error terms	Mean	SD		Error terms	Mean	SD	
Random Error*	0.00	6.86		Random Error*	0.00	0.71	
Residual*	0.00	4.64		Residual*	0.00	3.74	

The model terms are listed with the corresponding coefficients estimates (Coef. estimate), standard error (SE) and p-value. The factor ‘Group’ is defined as 1 if the mouse belongs to the ADR group, and as 0 otherwise. The error terms of the model are also reported. *Follows a normal distribution with mean zero and standard deviation (SD).

**Table 2:** Table summarising the associations in the ADR group between abnormal glomeruli (5w) and the four biomarkers: albuminuria at the maximum observed and at four weeks, FITC-Sinistrin half-life, IRDye clearance half-life, IRDye T_MAX_ delay.

Biomarker	Coef. estimate	SE	P-value
Albuminuria_MAX_ (mg/24 h)	0.04	0.05	0.45
Albuminuria_4w_ (mg/24 h)	0.2	0.15	0.21
FITC-sinistrin half-life_4w_ (min)	0.94	0.18	**0.001**
IRDye clearance half-life (s)	0.12	0.04	**0.02**
IRDye T_MAX_ delay (s)	0.16	0.03	**0.002**

The results included in this table were derived from the statistical models described in Table S5 by using *contrast analysis*. Each coefficient estimate indicates the change in percentage of abnormal glomeruli per unit change of the corresponding biomarker.

## References

[b1] Bussolati B. *et al.* Isolation of renal progenitor cells from adult human kidney. Am. J. Pathol. 166, 545–555, 10.1016/s0002-9440(10)62276-6 (2005). 15681837 PMC1602314

[b2] Harari-Steinberg O. *et al.* Identification of human nephron progenitors capable of generation of kidney structures and functional repair of chronic renal disease. EMBO Mol. Med. 5, 1556–1568, 10.1002/emmm.201201584 (2013). 23996934 PMC3799579

[b3] Lu J. *et al.* Discrete functions of M2a and M2c macrophage subsets determine their relative efficacy in treating chronic kidney disease. Kidney international 84, 745–755, 10.1038/ki.2013.135 (2013). 23636175

[b4] Ronconi E. *et al.* Regeneration of glomerular podocytes by human renal progenitors. J. Am. Soc. Nephrol. 20, 322–332, 10.1681/asn.2008070709 (2009). 19092120 PMC2637058

[b5] Kitiyakara C., Eggers P. & Kopp J. B. Twenty-one-year trend in ESRD due to focal segmental glomerulosclerosis in the United States. American journal of kidney diseases: the official journal of the National Kidney Foundation 44, 815–825 (2004). 15492947

[b6] Korbet S. M. Treatment of primary FSGS in adults. Journal of the American Society of Nephrology: JASN 23, 1769–1776, 10.1681/ASN.2012040389 (2012). 22997260

[b7] Jeansson M., Bjorck K., Tenstad O. & Haraldsson B. Adriamycin alters glomerular endothelium to induce proteinuria. J. Am. Soc. Nephrol. 20, 114–122, 10.1681/asn.2007111205 (2009). 19073829 PMC2615716

[b8] Lee V. W. & Harris D. C. Adriamycin nephropathy: a model of focal segmental glomerulosclerosis. Nephrology (Carlton, Vic.) 16, 30–38, 10.1111/j.1440-1797.2010.01383.x (2011). 21175974

[b9] Lee V. W. *et al.* Adriamycin nephropathy in severe combined immunodeficient (SCID) mice. Nephrol. Dial. Transplant 21, 3293–3298, 10.1093/ndt/gfl413 (2006). 16891644

[b10] Schock-Kusch D. *et al.* Transcutaneous assessment of renal function in conscious rats with a device for measuring FITC-sinistrin disappearance curves. Kidney Int. 79, 1254–1258 (2011). 21368744 10.1038/ki.2011.31

[b11] Schreiber A. *et al.* Transcutaneous measurement of renal function in conscious mice. Am. J. Physiol. Renal Physiol 303, F783–788 (2012). 22696603 10.1152/ajprenal.00279.2012

[b12] Fusellier M. *et al.* Influence of three anesthetic protocols on glomerular filtration rate in dogs. Am. J. Vet. Res. 68, 807–811, 10.2460/ajvr.68.8.807 (2007). 17669018

[b13] Mazze R. I., Cousins M. J. & Barr G. A. Renal effects and metabolism of isoflurane in man. Anesthesiology 40, 536–542 (1974). 4829722 10.1097/00000542-197406000-00006

[b14] Taruttis A., Morscher S., Burton N. C., Razansky D. & Ntziachristos V. Fast multispectral optoacoustic tomography (MSOT) for dynamic imaging of pharmacokinetics and biodistribution in multiple organs. PLoS ONE 7, doi: 10.1371 (2012). 10.1371/journal.pone.0030491 PMC3266258 22295087

[b15] Ntziachristos V. Going deeper than microscopy: The optical imaging frontier in biology. Nat. Methods 7, 603–614 (2010). 20676081 10.1038/nmeth.1483

[b16] Wang L. V. & Hu S. Photoacoustic Tomography: *In Vivo* Imaging from Organelles to Organs. Science 335, 1458–1462 (2012). 22442475 10.1126/science.1216210 PMC3322413

[b17] Lasagni L. *et al.* Notch activation differentially regulates renal progenitors proliferation and differentiation toward the podocyte lineage in glomerular disorders. Stem cells (Dayton, Ohio) 28, 1674–1685, 10.1002/stem.492 (2010). PMC2996085 20680961

[b18] Schock-Kusch D. *et al.* Transcutaneous measurement of glomerular filtration rate using FITC-sinistrin in rats. Nephrol. Dial. Transplant. 24, 2997–3001, 10.1093/ndt/gfp225 (2009). 19461009

[b19] Wang Y., Wang Y. P., Tay Y. C. & Harris D. C. Progressive adriamycin nephropathy in mice: sequence of histologic and immunohistochemical events. Kidney Int. 58, 1797–1804, 10.1046/j.1523-1755.2000.00342.x (2000). 11012915

[b20] Cowley A. W. Jr. *et al.* Progression of glomerular filtration rate reduction determined in conscious dahl salt-sensitive hypertensive rats. Hypertension 62, 85–90, 10.1161/hypertensionaha.113.01194 (2013). 23630946 PMC3806646

[b21] Schock-Kusch D. *et al.* Reliability of transcutaneous measurement of renal function in various strains of conscious mice. PLoS ONE 8, 1254–1258 (2013). 10.1371/journal.pone.0071519 PMC3747225 23977062

[b22] Zöllner F. G. *et al.* Simultaneous measurement of kidney function by dynamic contrast enhanced MRI and FITC-sinistrin clearance in rats at 3 tesla: Initial results. PLoS ONE 8, doi: 10.1371 (2013). 10.1371/journal.pone.0079992 PMC3832374 24260332

[b23] Ellery S., Cai X., Walker D., Dickinson H. & Kett M. M. Transcutaneous Measurement of Glomerular Filtration Rate in Small Rodents; Through the Skin for the Win? Nephrology (Carlton, Vic.) 10.1111/nep.12363 (2014). 25388805

[b24] Bonventre J. V., Vaidya V. S., Schmouder R., Feig P. & Dieterle F. Next-generation biomarkers for detecting kidney toxicity. Nature biotechnology 28, 436–440, 10.1038/nbt0510-436 (2010). PMC3033582 20458311

[b25] Dieterle F. *et al.* Urinary clusterin, cystatin C, beta2-microglobulin and total protein as markers to detect drug-induced kidney injury. Nature biotechnology 28, 463–469, 10.1038/nbt.1622 (2010). 20458316

[b26] Deman A. *et al.* Altered antioxidant defence in a mouse adriamycin model of glomerulosclerosis. Nephrology, dialysis, transplantation: official publication of the European Dialysis and Transplant Association - European Renal Association 16, 147–150 (2001). 10.1093/ndt/16.1.147 11209009

[b27] Zhang J. *et al.* Sex-related differences in mast cell activity and doxorubicin toxicity: a study in spontaneously hypertensive rats. Toxicologic pathology 42, 361–375, 10.1177/0192623313482778 (2014). 23531790

[b28] Lee V. W. *et al.* Adriamycin nephropathy in severe combined immunodeficient (SCID) mice. Nephrology, dialysis, transplantation: official publication of the European Dialysis and Transplant Association - European Renal Association 21, 3293–3298, 10.1093/ndt/gfl413 (2006). 16891644

[b29] Tan R. J., Zhou L., Zhou D., Lin L. & Liu Y. Endothelin receptor a blockade is an ineffective treatment for adriamycin nephropathy. PLoS One 8, e79963, 10.1371/journal.pone.0079963 (2013). 24265790 PMC3825716

[b30] Yasuda K. *et al.* Adriamycin nephropathy: a failure of endothelial progenitor cell-induced repair. The American journal of pathology 176, 1685–1695, 10.2353/ajpath.2010.091071 (2010). 20167859 PMC2843460

[b31] Endre Z. H., Pickering J. W. & Walker R. J. Clearance and beyond: the complementary roles of GFR measurement and injury biomarkers in acute kidney injury (AKI). Am J Physiol Renal Physiol 301, F697–707, 10.1152/ajprenal.00448.2010 (2011). 21753074

[b32] Murray P. T. *et al.* Potential use of biomarkers in acute kidney injury: report and summary of recommendations from the 10th Acute Dialysis Quality Initiative consensus conference. Kidney international 85, 513–521, 10.1038/ki.2013.374 (2014). 24107851 PMC4198530

[b33] Harrison T., Paproski R. J. & Zemp R. J. in SPIE BiOS. 82230S-82230S–82237 (International Society for Optics and Photonics).

[b34] Laufer J. *et al.* *In vivo* photoacoustic imaging of mouse embryos. Journal of biomedical optics 17, 061220, 10.1117/1.JBO.17.6.061220 (2012). 22734750

[b35] Nam S. Y., Ricles L. M., Suggs L. J. & Emelianov S. Y. *In vivo* ultrasound and photoacoustic monitoring of mesenchymal stem cells labeled with gold nanotracers. PLoS One 7, e37267, 10.1371/journal.pone.0037267 (2012). 22615959 PMC3353925

[b36] Dean-Ben X. L., Buehler A., Razansky D. & Ntziachristos V. Estimation of optoacoustic contrast agent concentration with self-calibration blind logarithmic unmixing. Physics in medicine and biology 59, 4785–4797, 10.1088/0031-9155/59/17/4785 (2014). 25097086

[b37] Deliolanis N. C. *et al.* Deep-tissue reporter-gene imaging with fluorescence and optoacoustic tomography: a performance overview. Molecular imaging and biology: MIB: the official publication of the Academy of Molecular Imaging 16, 652–660, 10.1007/s11307-014-0728-1 (2014). 24609633

[b38] Buehler A. *et al.* High resolution tumor targeting in living mice by means of multispectral optoacoustic tomography. EJNMMI Res 2, 14 (2012). 22464315 10.1186/2191-219X-2-14 PMC3337810

[b39] Jokerst J. V., Thangaraj M., Kempen P. J., Sinclair R. & Gambhir S. S. Photoacoustic imaging of mesenchymal stem cells in living mice via silica-coated gold nanorods. ACS nano 6, 5920–5930 (2012). 22681633 10.1021/nn302042y PMC3582222

[b40] Xiang L., Ahmad M., Hu X., Cheng Z. & Xing L. Label-free photoacoustic cell-tracking in real-time. X Acoust. Imaging Sens 1, 18–22 (2014).

[b41] Zhang X. *et al.* Resolvin D1 protects podocytes in adriamycin-induced nephropathy through modulation of 14-3-3beta acetylation. PLoS One 8, e67471, 10.1371/journal.pone.0067471 (2013). 23840712 PMC3696081

[b42] Kairaitis L. K., Wang Y., Gassmann M., Tay Y. C. & Harris D. C. HIF-1alpha expression follows microvascular loss in advanced murine adriamycin nephrosis. Am J Physiol Renal Physiol 288, F198–206, 10.1152/ajprenal.00244.2003 (2005). 15383400

[b43] Egger C. *et al.* Adriamycin-induced nephropathy in rats: functional and cellular effects characterized by MRI. J. Magn. Reson. Imaging, 10.1002/jmri.24603 (2014). 24596313

[b44] Preibisch S., Saalfeld S. & Tomancak P. Globally optimal stitching of tiled 3D microscopic image acquisitions. Bioinformatics 25, 1463–1465, 10.1093/bioinformatics/btp184 (2009). 19346324 PMC2682522

[b45] Licha K. *et al.* Hydrophilic cyanine dyes as contrast agents for near-infrared tumor imaging: synthesis, photophysical properties and spectroscopic *in vivo* characterization. Photochem. Photobiol. 72, 392–398 (2000). 10989611 10.1562/0031-8655(2000)072<0392:hcdaca>2.0.co;2

[b46] Hamann F. M. *et al.* Controlled modulation of serum protein binding and biodistribution of asymmetric cyanine dyes by variation of the number of sulfonate groups. Mol. Imaging 10, 258–269, 10.2310/7290.2011.00005 (2011). 21521558

