# Supplementary material for: Measures of kidney function by minimally invasive techniques correlate with histological glomerular damage in SCID mice with adriamycin-induced nephropathy
Source: Sci Rep. 2015 Sep 2;5:13601. doi: 10.1038/srep13601 (PMC4556979; doi:10.1038/srep13601)
Supplement: Supplementary Information [file srep13601-s1.pdf]

Measures of kidney function by minimally invasive techniques correlate with histological glomerular damage in SCID mice with adriamycin-induced nephropathy

Lauren Scarfe [a1], Aleksandra Rak-Raszewska [a1,8], Stefania Geraci [5], Darsy Darssan [2], Jack Sharkey [1], Jiaguo Huang [5], Neal C. Burton [6], David Mason [4], Parisa Ranjzad [7], Simon Kenny [1], Norbert Gretz [5], Raphaël Lévy [4], B. Kevin Park [3], Marta García-Fiñana [2], Adrian S. Woolf [7], Patricia Murray [1], Bettina Wilm [1*]

Supplementary Figures

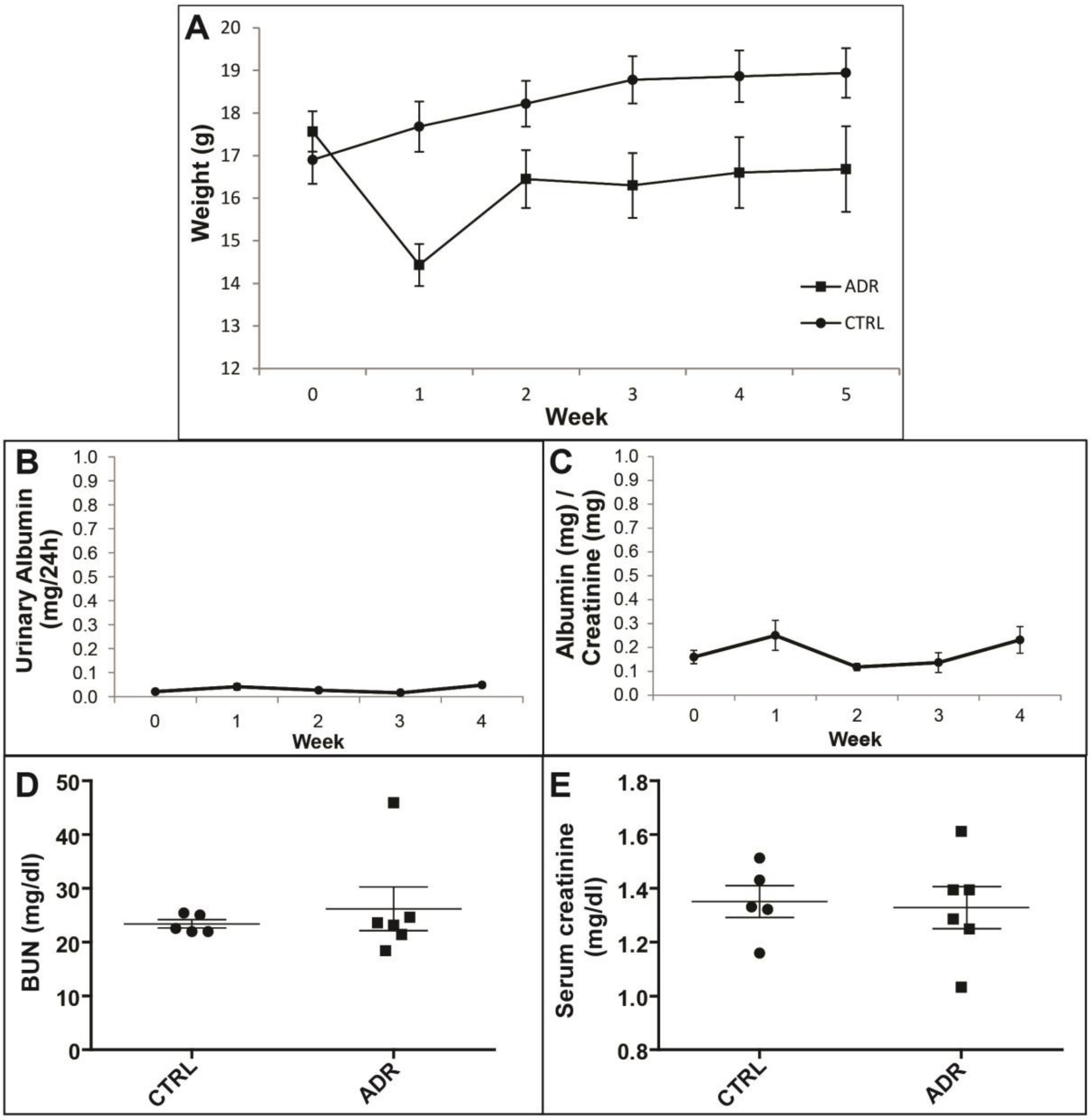

Supplementary Figure 1: A. Mean body weight measured weekly for 5 weeks. B, C. Mean 24h urinary albumin levels (B) and urinary albumin:creatinine ratio (C) in control animals only, measured weekly up to week 4. D, E. Blood urea nitrogen (BUN, D) and serum creatinine (E) in ADR-administered and control mice measured in serum collected via cardiac puncture immediately after sacrifice at week 5. Data points represent the group mean (A-C) or individual animals (D, E) (circles = control, *n*=5; squares = ADR-administered, *n*=6) and bars represent mean ± standard error.

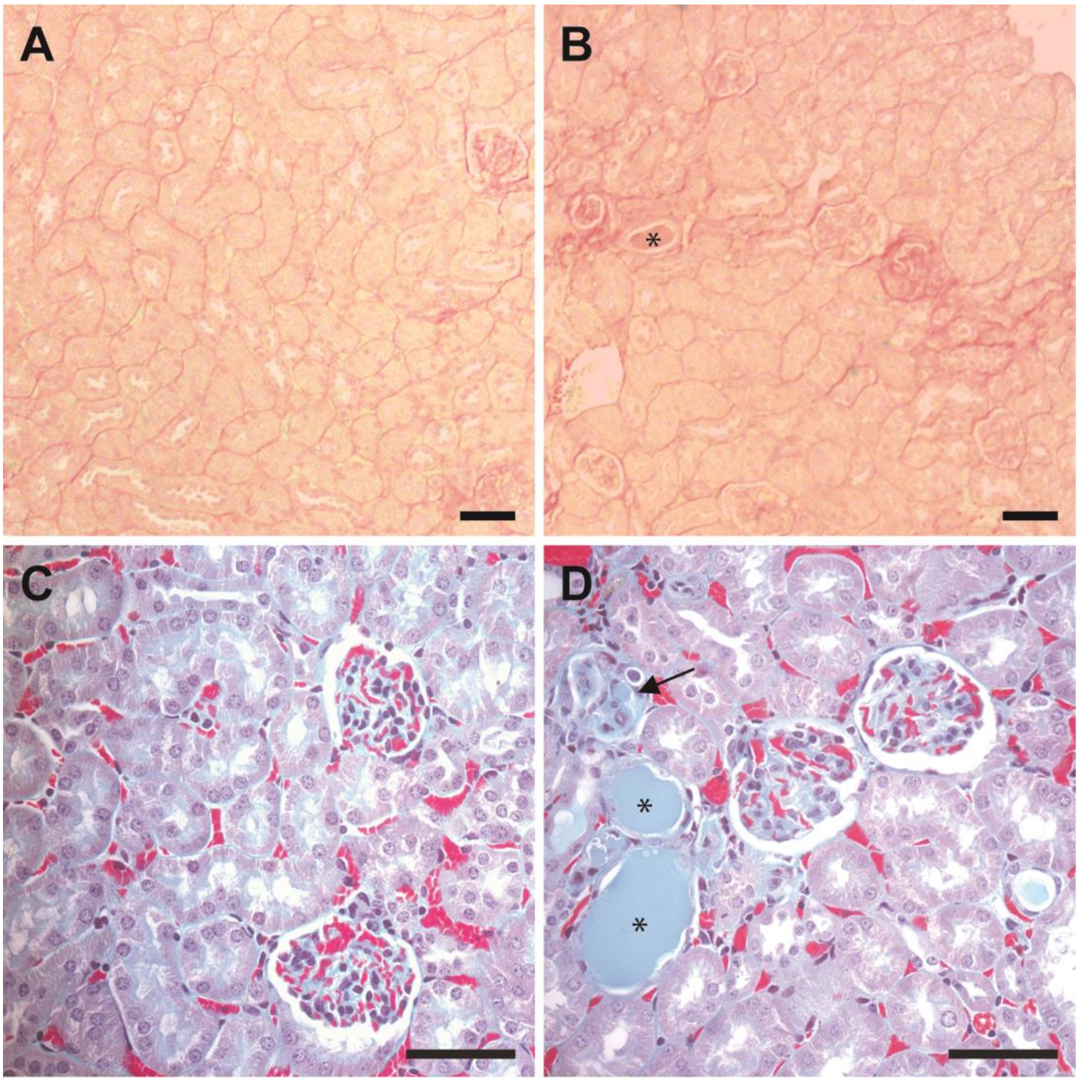

Supplementary Figure 2: Typical examples of normal (A, C) and slightly scarred (B, D) regions of the renal cortex imaged under polarised microscopy in Picro-Sirus Red-stained (A, B) or under bright field microscopy in Masson's Trichrome-stained (C, D) paraffin sections at week 5. Scale bars represent 50μm. Tubular casts (*) and scarred glomerulus (arrow) can be observed within the cortex of Picro-Sirius Red-stained and Masson's Trichrome-stained kidneys.

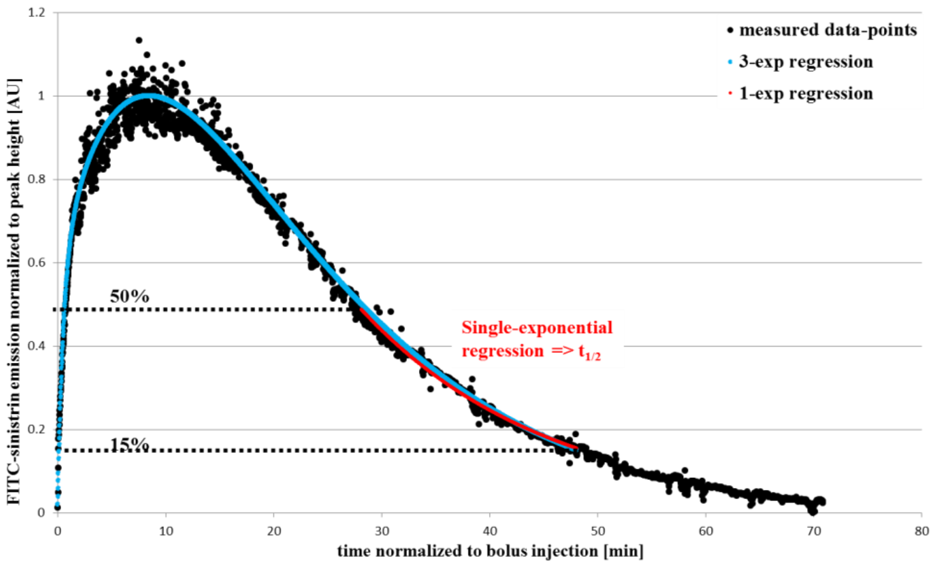

Supplementary Figure 3: Sample graph depicting FITC-sinistrin emission data points plotted against time after bolus injection to illustrate the way the 1-compartment fit is determined. The blue line shows the 3-exponential function, while the red line (1-exponential function) corresponds to the orange line in Figures 2A-D of the manuscript. Specifically, the 1-compartment fit was applied from 50% to 15% of the peak height (red curve) and $t_{1/2}$ was calculated as described in Schreiber et al., 2012 (PMID: 22696603). The reference peak height was recognized as the peak of the 3-exponential function that was preliminary fitted to the whole measured curve.

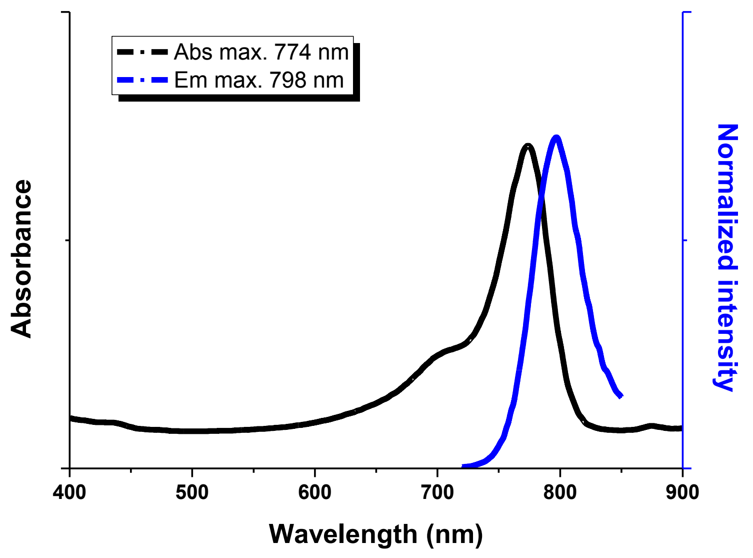

Supplementary Figure 4: Normalised absorption (black) and emission (blue) spectrum scan of 10 µmol IRDye in PBS. The absorption maximum is at 774 nm, while the emission maximum is at 798 nm.

		Urinary Albumin (mg/24h)					Urinary Albumin : Creatinine Ratio (mg/mg)					FITC-sinistrin half-life (minutes)				
	**Mouse ID**	**Week 0**	**Week 1**	**Week 2**	**Week 3**	**Week 4**	**Week 0**	**Week 1**	**Week 2**	**Week 3**	**Week 4**	**Week 0**	**Week 1**	**Week 2**	**Week 3**	**Week 4**
**ADR**	**A1**	0.02	16.10	59.49	34.11	23.23	0.20	123.61	176.84	153.16	176.00	8.14	x	15.05	14.25	19.80
	**A2**	0.01	4.30	45.53	32.30	9.06	0.14	35.34	176.21	226.65	46.02	10.60	10.10	14.90	10.76	11.00
	**A3**	0.04	11.08	85.16	66.98	32.19	0.16	97.98	271.34	192.71	114.56	12.19	14.11	14.10	17.47	27.19
	**A4**	0.01	0.74	37.21	139.00	16.19	0.13	3.40	180.82	372.47	90.78	10.64	14.67	17.67	13.18	16.91
	**A5**	0.04	0.87	28.34	54.40	44.71	0.19	3.91	82.12	260.72	132.80	12.43	x	11.34	x	15.75
	**A6**	0.01	1.16	46.57	149.05	35.89	0.14	6.78	140.94	629.59	92.70	8.10	13.32	15.11	32.11	16.82
	**Mean**	**0.02**	**5.71**	**50.38**	**79.31**	**26.88**	**0.16**	**45.17**	**171.38**	**305.88**	**108.81**	**10.35**	**13.05**	**14.69**	**17.55**	**17.91**
	**SE**	**0.01**	**2.63**	**8.14**	**21.18**	**5.39**	**0.01**	**21.57**	**25.18**	**71.57**	**17.94**	**0.77**	**0.83**	**0.83**	**3.46**	**2.19**
**CTRL**	**C1**	0.02	0.03	0.03	0.01	0.06	0.16	0.14	0.09	0.18	0.22	5.78	15.18	6.40	8.77	7.18
	**C2**	0.02	0.05	0.03	0.01	0.05	0.13	0.45	0.11	0.28	0.15	8.05	8.87	10.76	9.29	6.15
	**C3**	0.03	0.02	0.03	0.01	0.03	0.26	0.13	0.17	0.08	0.14	9.75	9.94	9.09	10.87	11.85
	**C4**	0.01	0.07	0.02	0.03	0.07	0.10	0.34	0.11	0.08	0.45	10.54	6.66	9.75	15.33	14.25
	**C5**	0.02	0.04	0.02	0.02	0.04	0.15	0.19	0.10	0.06	0.20	6.62	7.78	11.00	8.70	13.47
	**Mean**	**0.02**	**0.04**	**0.03**	**0.02**	**0.05**	**0.16**	**0.25**	**0.12**	**0.14**	**0.23**	**8.15**	**9.69**	**9.40**	**10.59**	**10.58**
	**SE**	**0.01**	**0.01**	**0.01**	**0.01**	**0.01**	**0.03**	**0.06**	**0.01**	**0.04**	**0.06**	**0.90**	**1.48**	**0.83**	**1.25**	**1.65**

Supplementary Table 1A: Table summarising the individual values of all longitudinal parameters measured in adriamycin (ADR)-administered and control (CTRL) mice from weeks zero to four. The mean and standard error (SE) for ADR-administered and control groups are shown. x = data unsuitable for analysis as the kinetics of the curves obtained at these time points were significantly affected by movement artefacts.

	Mouse ID	T_{MAX} delay (s)	Excretion half-life (cortex) (s)	% Abnormal glomeruli	% PSR staining	% Fibrillar collagen	Serum creatinine (mg/dl)	Blood urea nitrogen (mg/dl)
		Week 5	Week 5	Week 5	Week 5	Week 5	Week 5	Week 5
ADR	**A1**	143.99	220.20	22.22	2.33	1.06	1.39	23.60
	**A2**	29.02	154.80	12.50	2.28	3.24	1.03	18.42
	**A3**	116.01	189.60	29.14	3.95	1.48	1.61	45.95
	**A4**	96.01	172.20	26.02	1.02	0.09	1.25	21.42
	**A5**	112.02	141.60	23.81	1.70	1.73	1.39	24.65
	**A6**	80.02	167.40	19.57	2.34	2.32	1.29	23.18
	**Mean**	**96.18**	**174.30**	**22.21**	**2.27**	**1.65**	**1.33**	**26.21**
	**SE**	**16.02**	**11.32**	**2.35**	**0.40**	**0.44**	**0.08**	**4.05**
CTRL	**C1**	31.99	159.00	5.13	1.58	1.53	1.32	22.56
	**C2**	32.00	148.80	0.00	1.74	1.63	1.43	21.99
	**C3**	47.99	65.40	0.00	2.79	2.62	1.16	25.07
	**C4**	15.98	119.40	0.00	1.77	2.05	1.51	25.43
	**C5**	64.01	151.80	1.30	1.08	1.03	1.33	21.99
	**Mean**	**38.40**	**128.88**	**1.29**	**1.79**	**1.77**	**1.35**	**23.41**
	**SE**	**8.16**	**17.25**	**0.99**	**0.28**	**0.27**	**0.06**	**0.76**

Supplementary Table 1B: Table summarising the individual values of end-point measures in adriamycin (ADR)-administered and control (CTRL) mice 5 weeks after the administration of adriamycin. The mean and standard error (SE) for ADR-administered and control groups are shown.

	Normally Distributed Data assumed?	Statistical test	Test value	P-value	Degrees of Freedom
**T_{MAX} delay (s)**	Yes	Two-sample t-test assuming unequal variance	T = 3.21	0.015	7
**Excretion half-life (cortex) (s)**	Yes	Two-sample t-test assuming unequal variance	T = 2.2	0.064	7
**% Abnormal glomeruli**	No	Wilcoxon rank sum test with continuity correction	W = 30	0.008	
**% PSR staining**	Yes	Two-sample t-test assuming unequal variance	T = 0.95	0.366	9
**% Fibrillar collagen**	Yes	Two-sample t-test assuming unequal variance	T = -0.25	0.809	9
**Serum creatinine (mg/dl)**	Yes	Two-sample t-test assuming unequal variance	T = -0.23	0.821	8
**Blood urea nitrogen (mg/dl)**	No	Wilcoxon rank sum test with continuity correction	W = 14	0.927	

Supplementary Table 2: Table summarising the statistical analyses used to assess if there was a statistically significant difference between the adriamycin (ADR)-administered and control groups for parameters measured at week 5 only. Normality tests were applied to check the assumption that the data come from a normal distribution; when the normality hypothesis was rejected a non-parametric test was applied. No adjustment for multiple comparisons was made.

	Week 0	Week 1	Week 2	Week 3	Week 4	Week 5
**Weight change**		p<0.001	p<0.001	p<0.001	p<0.001	p<0.001
**24h Albumin**	p=0.999	p=0.194	p=0.001	p=0.004	p=0.001	
**Alb:Cr**	p=0.999	p=0.223	p=0.001	p=0.001	p=0.001	
**FITC-sinistrin half-life**	p=0.510	p=0.326	p=0.029	p=0.041	p=0.005	

Supplementary Table 3: Table summarising the results of mixed-design ANOVA models carried out to assess the significance of comparisons between adriamycin-administered mice and time-matched controls at multiple time points.

		**10µmol IRDye**			**PBS**			**IRDye mean – PBS mean**
**Rat Plasma**	Abs	0.2046	0.202	0.2051	0.1673	0.1663	0.1691	0.0364
	Mean	0.2039			0.1675			
**PBS**	Abs	0.0595	0.058	0.054	0.0428	0.042	0.0416	0.0151
	Mean	0.0572			0.0421			
**PPB**	[A(plasma) - A(PBS)] / [A(plasma) + A(PBS)] * 100 = [0.0364 – 0.0151] / [0.0364 + 0.0151] * 100							**41%**

Supplementary Table 4: IRDye protein plasma binding (PPB) determined by measuring the UV absorption (A) at 774nm of IRDye or phosphate buffered saline (PBS) incubated with rat plasma or PBS for 24 hours.

Percentage of glomerular histological damage (5w) and its association with:

(i) maximum albuminuria (mg/24h)

Term	Coef. estimate	SE	P-value
Intercept (%)	1.29	2.08	0.55
Group	17.65	4.97	0.008
Albuminuria_{MAX} x Group (mg/24h)	0.04	0.05	0.45
Error terms	Mean	SD	
Residual*	0.00	4.64	

(ii) albuminuria at 4w (mg/24h)

Term	Coef. estimate	SE	P-value
Intercept (%)	1.29	1.95	0.53
Group	15.57	4.76	0.01
Albuminuria_{4w} x Group (mg/24h)	0.2	0.15	0.21
Error terms	Mean	SD	
Residual*	0.00	4.35	

(iii) FITC-sinistrin half-life at 4w (min)

Term	Coef. estimate	SE	P-value
Intercept (%)	5.15	3.23	0.15
FITC_{4w} half-life (min)	-0.36	0.3	0.28
FITC_{4w} half-life x Group (min)	1.3	0.18	**0.001**
Error terms	Mean	SD	
Residual*	0.00	3.00	

(iv) IRDye excretion half-life at 5w (s)

Term	Coef. estimate	SE	P-value
Intercept (%)	0.92	7.01	0.9
Excretion half-life (s)	0.005	0.05	0.93
Excretion half-life x Group (s)	0.116	0.02	**0.001**
Error terms	Mean	SD	
Residual*	0.00	4.74	

(v) IRDye T_{MAX} delay at 5w (s)

Term	Coef. estimate	SE	P-value
Intercept (%)	6.27	3.29	0.09
T_{MAX} delay (s)	-0.11	0.08	0.23
T_{MAX} delay x Group (s)	0.27	0.06	**0.003**
Error terms	Mean	SD	
Residual*	0.00	4.03	

Supplementary Table 5: Mathematical representation (multiple regression models) of the association between abnormal glomeruli at week 5 and (i) maximum albuminuria, (ii) albuminuria at week 4, (iii) FITC-Sinistrin half-life at week 4, (iv) IRDye excretion half-life in the cortex at week 5, and (v) IRDye T_{MAX} at week 5. The factor Group is defined as 1 if the mouse belonged to the adriamycin (ADR) group, and as 0 otherwise. Goodness-of-fit was assessed for each model using the R^2 values: Model (i) R^2 = 0.84, (ii) R^2 = 0.86, (iii) R^2 = 0.93, (iv) R^2 = 0.84, (v) R^2 = 0.88. *Follows a normal distribution with mean zero and standard deviation (SD).

Supplementary Movie 1.

Movie 1

Movie showing the clearance of IRDye (20nmol) through the kidney of an adriamycin (ADR)-administered mouse at week 5. IRDye was administered to the mouse through a tail vein cannula at 4.8 min and can be first seen in the renal cortex before it clears through the cortex and collects in the renal pelvis. Videos are displayed at 3 frames per second (1 frame is equal to approximately 15 seconds).

Supplementary Movie 2.

Movie 2

Movie showing the clearance of IRDye (20nmol) through the kidney of a control mouse at week 5. IRDye was administered to the mouse through a tail vein cannula at 4.4 minutes and can be first seen in the renal cortex before it clears through the cortex and collects in the renal pelvis. Videos are displayed at 3 frames per second (1 frame is equal to approximately 15 seconds).
